# circ_0001274 Competitively Binds miR-143-3p to Upregulate VWF Expression to Improve Acute Traumatic Coagulopathy

**DOI:** 10.1155/2023/9650323

**Published:** 2023-01-31

**Authors:** Yong Luo, Shan Liu, Qing Li, Yonghu Zhang, Yong Fu, Ning Yang, Jian Zeng, Taifa Tan, Jun Hu, Fang Li, Liang Zeng, Wenchao Zhang, Zhanchen Liao, Kai Wu, Yu Hu, Zhigang Luo, Jian Peng

**Affiliations:** ^1^Hengyang Medical School, University of South China, Hengyang 421001, China; ^2^Trauma Center, Hengyang Medical School, The Second Affiliated Hospital, University of South China, Hengyang 421001, Hunan Province, China; ^3^Department of Rehabilitation Medicine, Xiangya Hospital, Central South University, Changsha 4140000, China; ^4^National Clinical Research Center for Geriatric Diseases, Changsha 4140000, China; ^5^Center for Experimental Medical Research, Third Xiangya Hospital, Central South University, Changsha 410013, China; ^6^Department of General Surgery, Xiangya Hospital, Central South University, Changsha 410008, China

## Abstract

Accumulating evidence has noted the circRNA-microRNA- (circRNA-miRNA-) mRNA competing endogenous RNA (ceRNA) regulatory network in disease development and progression. The current study explored the ceRNA network in acute traumatic coagulopathy (ATC). Potential ATC-related genes were screened, and upstream miRNAs and circRNAs of VWF (the candidate target) were assayed through database searching and high-throughput sequencing technology. circ_0001274/miR-143-3p/VWF ceRNA regulatory network was constructed and validated. The expression of circ_0001274/miR-143-3p/VWF was determined in the peripheral blood samples from ATC patients and ATC mouse models. Online database and circRNA sequencing analysis results identified VWF as a key gene in ATC as supported by assays and that VWF was lowly expressed in ATC patients and mice. Further experiments demonstrated that miR-143-3p could target and inhibit VWF, and circ_0001274 could competitively sponge miR-143-3p. Functionally, circ_0001274 could competitively sequester miR-143-3p to upregulate VWF expression, potentially improving ATC. Our study highlights the critical role of circ_0001274/miR-143-3p/VWF axis in improving ATC.

## 1. Introduction

Trauma is a leading cause of death and permanent disability among adults, and more than 25-35% of trauma patients occur with coagulopathy at the time of admission to the emergency department [1]. Acute traumatic coagulopathy (ATC) is the failure of coagulation homeostasis that can suddenly occur after traumatic injury, hemorrhage, and shock [2]. ATC responds quickly to tissue damage and hemorrhagic shock and is exacerbated by hypothermia and acidosis, as well as hypocoagulable fluid resuscitation [3]. Evidence revealed that activation of protein C, depletion of fibrinogen, disruption of endothelial glycocalyx, and platelet dysfunction were the identified mechanisms for ATC [4]. It is a challenge of early diagnosis of coagulopathy in the patients with bleeding trauma due to no effective assays for reliably ensuring aberrant haemostasis in the acute phase [5].

Von Willebrand Factor (VWF), a large glycoprotein, synthesized in endothelial cells and megakaryocytes, plays essential roles in haemostasis and thrombosis [6–8]. The lack of VWF has been shown to result in enhanced blood vessel formation both constitutively and following ischemia and leads to damaged arteriogenesis and angiogenesis after femoral artery ligation [9]. Accumulating evidence indicates that microRNAs (miRNAs) act as regulators in the hemostatic system through working with the mRNAs that encode proteins engaged in coagulation [10]. miR-143/145, a vascular-enriched miRNA cluster, is highly studied in vascular biology and in cardiovascular diseases [11, 12]. Moreover, circRNAs are reported to serve as miRNA sponges, which are proved to negatively regulate miRNAs by miRNA molecules [13]. Previous studies confirm that circRNAs are also found in body fluids such as human blood and are clearly enriched in platelets [14, 15]. The function of circ_0001274 in diseases and cancers was rarely studied. On a recent study, it demonstrated that circ_0001274 was capable of repressing ovarian granulosa cell proliferation through regulation of CDK4 [16]. However, the critical role of circ_0001274 in ATC remains unclear. Through database search and analysis, we speculated that circ_0001274 affected miR-143-3p through competitive adsorption and then affected the target gene VWF of miR-143-3p, so as to identify VWF as a candidate gene affecting ATC. Here, we tried to explore the underlying mechanism concerning circ_0001274-related ceRNA network in ATC through established ATC mouse models.

## 2. Material and Methods

### 2.1. Ethics Statement

Our study was approved by the Ethics Committee of The Second Hospital of the University of South China. Informed written consent was provided from each participant prior to the study. All experiments abided by the Declaration of Helsinki. Animal experimentations were strictly designed and completed in the light of the Guide for the Care and Use of Laboratory Animal by the US National Institutes of Health.

### 2.2. circRNA and miRNA Sequencing

The peripheral blood samples of 3 ATC patients and 3 normal healthy persons in The Second Hospital of the University of South China were collected, and total RNA was isolated by TRIzol reagent (Invitrogen). NanoDrop ND-1000 spectrophotometer (Thermo Fisher Scientific) was applied to measure RNA sample concentration as the OD260/280 ratio. The integrity of RNA was checked by agarose gel electrophoresis with 28S: 18S ratio ≥ 1.5. circRNA sequencing was performed using the Illumina NEBNext Ultra RNA Library Prep Kit (NEB, USA). An Illumina TruSeq® Small RNA Library Prep Kit was used as the miRNA sequencing library. A total of 5 *μ*g RNA was used for each sample. RNA sequencing library quantification kit (KAPA Biosystems) was applied for quantification and quality inspection. Paired-end sequencing was completed on the Illumina NextSeqCN500 sequencer. circRNA sequencing was performed on the peripheral blood samples of 3 ATC patients and 3 normal healthy people to screen the differentially expressed circRNAs in the peripheral blood samples of ATC patients.

### 2.3. Bioinformatics Analysis

ATC-related microarray data was screened from GeneCards dataset. The interaction network of target genes was harvested through the STRING database with the species condition limited to “Homo sapiens”. Through the FunRich 3.1.3 software, the cell functions and signal pathways that were mainly affected by potential target genes were analyzed. miRWalk database was adopted to predict the upstream miRNA of VWF. Through the StarBase database, the upstream circRNA of miRNA was predicted. The online database screening results and the circRNA sequencing results were intersected to construct a circRNA-miRNA-mRNA regulatory network (ceRNA network) involved in the occurrence of ATC.

### 2.4. Clinical Sample Collection

A total of 87 patients with acute trauma admitted to The Second Hospital of the University of South China from June 2018 to June 2020 were selected as the research objects. The criteria for case selection included (1) patients with trauma caused by various reasons; (2) patients arrived at the hospital within 24 hours after injury; (3) patients with injury severity score (ISS) > 16; and (4) patients' completed blood coagulation function testing and first treatment in the emergency department or admission (including death before discharge). The exclusion criteria were (1) pregnant women; (2) patients with old thrombosis, hemophilia, and other coagulation abnormalities; (3) patients who had long term use of anticoagulant or antiplatelet drugs; (4) patients who received blood transfusion before going to the hospital; (5) patients with simple burn; and (6) patients with simple head injury. In order to avoid deviation, the ISS score of all ATC patients was completed by the author based on the previously published study [17].

In this study, the 24 h peripheral venous blood INR > 1.2 was used as the diagnostic criteria for ATC. According to whether the INR was greater than 1.2, they were randomized into ATC group (*n* = 39) and non-ATC group (*n* = 48). The two groups of patients were compared with indicators such as gender, age, injury mechanism, injury location distribution, ISS score, vital signs and postadmission examination results, and blood transfusion within 24 h (Table S1). A total of 5 ml of peripheral blood samples were harvested from the two groups, and anticoagulated whole blood was centrifuged at 4°C within 6-8 h. The plasma was separated and quickly frozen into blocks below -30°C, which was called fresh frozen plasma. The fresh frozen plasma was stored at -80°C for subsequent experimentations.

### 2.5. Mice Model Establishment

A total of 96 C57BL/6J mice (male, age: 8 weeks, weight: 20 g-25 g) purchased from Hunan SJA Laboratory Animal Co., Ltd., was raised in an SPF animal laboratory. They were reared in separate cages, with a humidity of 60%~65%, a temperature of 22~25°C, and a 12-hour light and dark cycle. The experiments started after a 1-week acclimation period. The modeled mice were treated with saline, VWF, NC inhibitor, miR-143-3p inhibitor, sh-VWF, oe-NC, oe-circ_0001274, or sh-NC. Sham-operated mice (*n* = 12) were used as control.

Construction of the ATC models was performed as previously described [18–20]. Mean arterial pressure (MAP) in mice was 125 mmHg. The blood pressure of the mice was monitored, and the arterial blood was drawn after the arterial catheterization, at the time of shock (*T* = 0 min), 60, 180, and 240 min after the shock to measure the prothrombin time (PT) and activated partial thromboplastin time (APTT) in the peripheral blood using Thrombotimer (Behnk Elektronik, Germany) [20]. After 240 min, 2 mL of blood was drawn from the arterial catheter to prepare peripheral blood which was centrifuged at 1000 g for 15 min at 4°C. The supernatant was collected and stored at -80°C.

Except for the sham group and the ATC group, the mice in the other groups were treated by intraperitoneal injection of corresponding adenoviruses immediately after the trauma operation was completed. The adenovirus used in the experiment was provided by the Shanghai Geneland Biotech Co., Ltd. (adenovirus titer was 1 × 10^11^ PFU, and the injection volume per mouse was about 50 *μ*L). Mice in the ATC+VWF group received a single dose of VWF concentrate (3 U VWF: RCo/mouse) (Humate-P, CSL Behring) for intraperitoneal injection immediately after the trauma operation was completed. Surgery and intraperitoneal injection were performed in a sterile environment.

### 2.6. Enzyme-Linked Immunosorbent Assay (ELISA)

The level of VWF (VWF:Ag) antigen in peripheral blood was detected with an ELISA kit (DY2764-05, R&D Systems Inc., USA). The level of activated protein C (aPC) in peripheral blood was detected with an ELISA kit (ARB13146, Beijing Biolab Technology Co., Ltd., China).

### 2.7. Thrombelastography (TEG)

TEG 5000 Thromboelastogram Coagulation Analyzer (American Haemoscope III) was applied for detection. Briefly, an appropriate amount of sodium citrate liquid was added to the collected blood for anticoagulation (900 *μ*L of whole blood: 100 *μ*L of 4% sodium citrate). The blood sample was mixed and allowed to stand for 30 min to facilitate the mixing of citric acid in the blood sample. Afterwards, 340 *μ*L of this sample was dropped into a TEG cuvette (containing 0.2 M CaCl_2_), and the cuvette was placed in a thromboelastogram detector at 37°C for detection. The related indicators of TEG are shown in Table S2.

### 2.8. Cell Culture and Transfection

HEK293T cell lines were purchased from the American Type Culture Collection (ATCC, USA). Cells were cultured in DMEM (10569044, Gibco, USA) containing 10% fetal bovine serum (10099141, Gibco, USA), 2 mM L-glutamine (Sigma-Aldrich, St. Louis, MO, USA), 100 U/mL penicillin, and 100 *μ*g/mL streptomycin and incubated in a 37°C, 5% CO_2_ incubator.

Human umbilical vein endothelial cell lines (HUVECs) were purchased from Shanghai Honsun Biological Technology Co., Ltd. HUVECs (4 × 10^5^ cells/well) were inoculated into a 6-well cell culture plate. When the cell confluence reached 70-80%, cells were transfected with oe-NC (NC for overexpression of circ_0001274), oe-circ_0001274 (overexpression of circ_0001274), sh-NC (NC for silencing of circ_0001274), sh-circ_0001274 (silencing of circ_0001274), mimic-NC (NC for miR-143-3p mimic), miR-143-3p mimic, inhibitor-NC (NC for miR-143-3p inhibitor), and miR-143-3p inhibitor using Lipofectamine 2000 (11668-019, Invitrogen, CA, USA). All transfection sequences (Table S3) and plasmids were provided from Shanghai GenePharma Co., Ltd.

### 2.9. Reverse Transcription Quantitative Polymerase Chain Reaction (RT-qPCR)

Total RNA was isolated from cells using TRIzol reagent (16096020, Thermo Fisher Scientific, USA). The total RNA was reverse-transcribed into cDNA by using PrimeScript reverse transcriptase kit (Takara, Dalian, China) and PrimeScript miRNA reverse transcriptase kit (Takara, Dalian, China). RT-qPCR (Q511-02, Vazyme Biotech, Nanjing, China) was carried out according to the instructions. The primer sequence was designed and provided by Shanghai Sangon Biotech Co., Ltd. (Sangon Biotech, Shanghai, China). The primer sequence is described in Table S4. The 2^−ΔΔCt^ method was used to quantify relative expression of target genes [21].

### 2.10. Western Blot Analysis

Total protein extracts were subjected to 10% sodium dodecyl sulfate-polyacrylamide gel electrophoresis and transferred onto a polyvinylidene fluoride membrane (IPVH85R, Millipore, MA, USA). After 1 h of blocking with 5% BSA, the membrane was incubated with the corresponding primary antibodies, rabbit monoclonal antibody VWF (ab174290, 1 : 1000, Abcam, Cambridge, UK) and GAPDH (internal reference, ab181602, 1 : 10000, Abcam). After that, the membrane was incubated with HRP-labeled secondary antibody IgG (ab6721, 1 : 5000, Abcam) for 2 h. The signal was detected by the ECL kit (Bio-Rad). The quantitative analysis of protein was carried out by ImageJ software (V1.48, National Institutes of Health, USA).

### 2.11. Dual-Luciferase Reporter Gene Assay

The luciferase experiment was used to verify whether VWF was the direct target gene of miR-143-3p. The constructed VWF-WT and VWF-MUT were cotransfected with miR-143-3p mimic and negative control into HEK293 cells. Method for dual-luciferase activity detection was provided by Promega. The same method was applied to verify the targeting relationship between circ_0001274 and miR-143-3p.

### 2.12. RNA-Binding Protein Immunoprecipitation (RIP) Assay

RIP kit (#17-700, Millipore, USA) was applied to test the binding of circ_0001274 to miR-143-3p [22]. The antibodies used in RIP were rabbit antihuman AGO2 (2 *μ*g, Abcam, ab32381) and rabbit antihuman IgG (2 *μ*g, ab109489, Abcam, used as NC).

### 2.13. RNA Pull-Down Assay

HUVECs overexpressing circ_0001274 were transfected with biotinylated WT miR-143-3p and biotinylated MUT miR-143-3p (50 nM each) for pull-down assay [22]. The bound RNA was purified by TRIzol, and the enrichment of circ_0001274 was detected by RT-qPCR.

### 2.14. Fluorescence *In Situ* Hybridization (FISH)

FISH technology was used to identify the location of circ_0001274 and miR-143-3p in cells by RiboTM lncRNA FISH Probe Mix (Red) (Ruibo Bio, China) [22]. Five different fields of view under the confocal laser microscope (Leica, Germany) were selected to observe and take pictures.

### 2.15. Statistical Analysis

Data analysis was performed using the SPSS 21.0 (IBM-SPSS Inc., Chicago, IL, USA). All measurement data are presented as mean ± standard deviation. Two sets of data of unpaired design were compared using unpaired *t*-test. Data comparisons among multiple groups were performed using one-way analysis of variance (ANOVA) with Tukey's post hoc test. Data comparisons at different time points were performed by repeated measures ANOVA with Tukey's post hoc test. Values of *p* < 0.05 were considered significant.

## 3. Results

### 3.1. The Significance of VWF in the Occurrence of ATC

**Figure 1:**
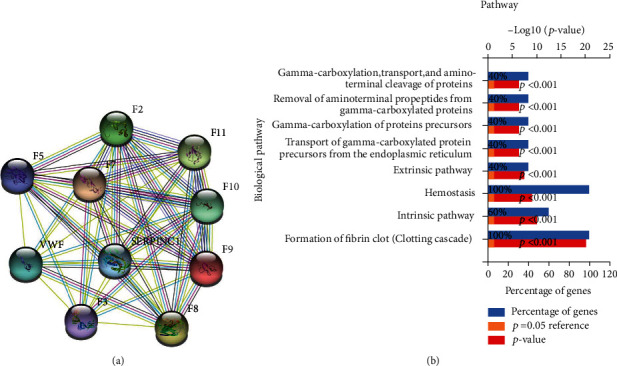
Online database screening of target genes involved in the occurrence of ATC. (a) Interaction network diagram of 10 disease interaction genes (nodes represent proteins, and edges indicate interrelationships between proteins). (b) Functional enrichment analysis diagram of 10 genes.

The top 10 genes related to coagulation dysfunction (F2, F8, F9, F5, F7, VWF, F11, F3, F10, and SERPINC1) were retrieved from the GeneCards database. The protein interaction network diagram of these 10 candidate target genes was further obtained through the STRING database (Figure 1(a)). The functional enrichment analysis of these 10 candidate genes by FunRich 3.1.3 software found that the biological functions of these 10 candidate genes were mainly enriched in coagulation function (Figure 1(b)), suggesting that these 10 candidate genes may be closely related to the occurrence of ATC. It has been reported that VWF not only acts importantly in trauma haemostasis but also in secondary inflammation and coagulation disorders [23]. Therefore, we speculated that VWF may be a key gene engaged in ATC.

### 3.2. VWF Is Lowly Expressed in Peripheral Blood of ATC Patients and Mice

**Figure 2:**
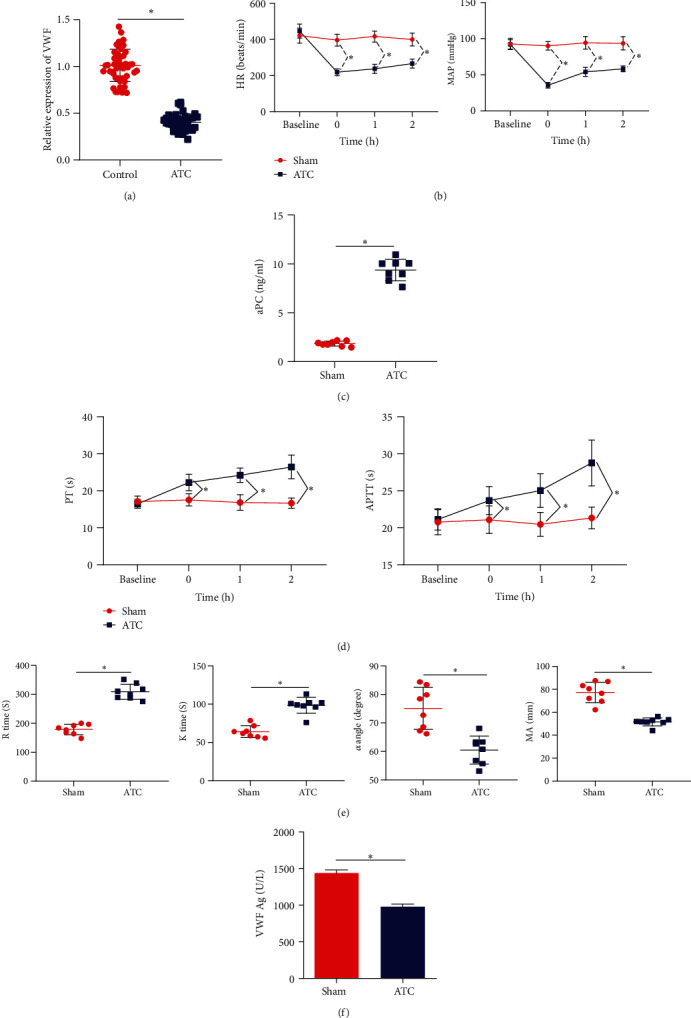
Lowly expressed VWF in peripheral blood of ATC patients and mice. (a) ELISA to detect the expression level of VWF in the peripheral blood of ATC patients (non-ATC: *n* = 48, ATC: *n* = 39). (b) Comparison of mean arterial pressure and heart rate of mice in each group. (c) ELISA to detect level of activated protein C in peripheral blood of mice in each group. (d) Comparison of prothrombin time and activated partial thromboplastin time of mice in each group. (e) Comparison of TEG test results of mice in each group. (f) ELISA to detect the expression level of VWF in the peripheral blood of mice in each group. *n* = 8 mice for each group. ^∗^*p* < 0.05.

ELISA detection of the expression level of VWF in the peripheral blood of 87 ATC patients showed that VWF expression in the peripheral blood of ATC patients was lower than that of non-ATC patients (Figure 2(a)). To further explore the effect of VWF on ATC, we constructed an ATC mouse model. We found no significant difference in comparison of the MAP and heart rate (HR) of mice in the ATC group and the sham group, but the MAP and HR of ATC mice 0, 1, and 2 h after shock were significantly lower than those in sham-operated mice, showing significant difference; and after shock, HR and MAP levels recovered slightly with time in the detection time range (Figure 2(b)). ELISA results showed that 1 h after trauma, the concentration of aPC in the peripheral blood of the ATC mice was increased significantly (Figure 2(c)). Besides, we observed increased PT and APTT in the ATC mice (Figure 2(d)). Moreover, TEG results revealed that the reaction time (*R* time) and coagulation time (*K* time) of the mice in the ATC group were increased, while the *α* angle and maximum amplitude (MA) were reduced, indicating that the ATC mice had slowed production of thrombin and weakened thrombosis ability due to traumatic shock (Figure 2(e)). The above results validated the successful establishment of an ATC mouse model. Further, the determination of VWF expression in peripheral blood of mice also showed the same results as found in the peripheral blood of ATC patients (Figure 2(f)).

### 3.3. Exogenous VWF Improves the Coagulation Function of ATC Mice

**Figure 3:**
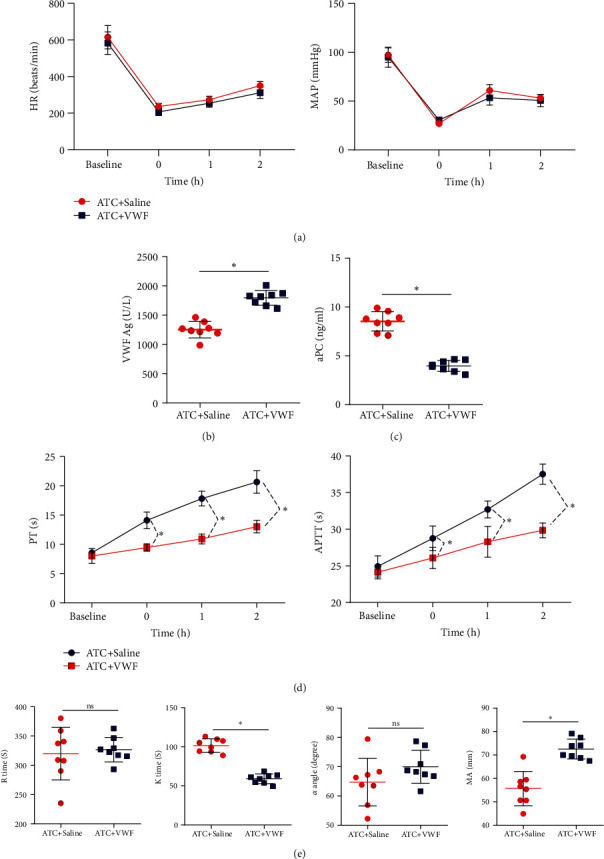
VWF improves the coagulation function of ATC mice. (a) Comparison of mean arterial pressure and heart rate of mice in each group. (b) ELISA to detect the expression level of VWF in the peripheral blood of each group of mice. (c) ELISA to detect activated protein C in the peripheral blood of each group of mice. (d) Comparison of prothrombin time and activated partial thromboplastin time of mice in each group. (e) Comparison of TEG test results of mice in each group. *n* = 8 mice for each group. ^∗^*p* < 0.05.

To further explore the effect of VWF on ATC, we constructed an ATC mouse model and intraperitoneally injected it with VWF (3 U VWF: RCo/mouse, Humate-P, CSL Behring) derived from human peripheral blood or physiological saline. The HR of ATC mice injected with saline or VWF increased slightly after shock, but there was no significant difference in HR between them; after shock, the MA*P* value of ATC mice injected with saline or VWF increased first then decreased with no significant difference observed (Figure 3(a)).

ELISA results indicated that ATC mice injected with VWF showed increased VWF levels in peripheral blood and decreased aPC after 1 h of trauma (Figures 3(b) and 3(c)). Determination of PT and APTT showed that ATC mice injected with VWF had reduced PT and APTT (Figure 3(d)). TEG analysis showed that ATC mice injected with VWF had decreased *K* time but elevated MA, while no significant difference was observed in *R* time and *α* angle (Figure 3(e)). The above findings demonstrated that exogenous VWF could improve the coagulation function of ATC mice.

### 3.4. miR-143-3p Targets and Inhibits VWF

**Figure 4:**
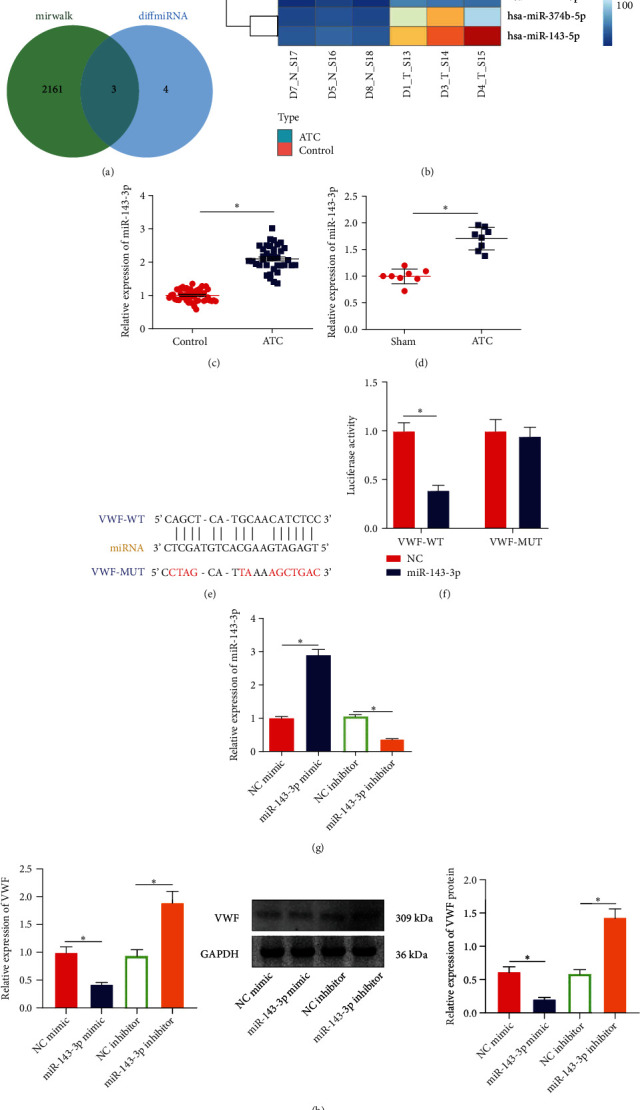
miR-143-3p targets VWF. (a) Venn diagram of the upstream regulatory miRNAs of VWF predicted by miRWalk and the differential miRNAs in ATC. (b) Heat map of differentially expressed miRNAs in ATC. (c) RT-qPCR to detect the expression of miR-143-3p in the peripheral blood of ATC patients (non-ATC: *n* = 48, ATC: *n* = 39). (d) RT-qPCR to detect the expression of miR-143-3p in the peripheral blood of mice in each group. (e) The binding site of miR-143-3p and VWF predicted by StarBase database. (f) Dual luciferase assay to detect the binding of miR-143-3p and VWF. (g) RT-qPCR to detect the expression of miR-143-3p and VWF in each group of cells. (h) Western blot to detect the protein expression of VWF. *n* = 8 mice for each group. ^∗^*p* < 0.05. All cell experiments were repeated for three times.

We further predicted the upstream miRNA of VWF through the online database miRWalk, and 2164 miRNAs were obtained. Then, intersection was performed between the obtained 2164 miRNAs and the differentially expressed miRNAs screened by the second-generation high-throughput sequencing of miRNAs, and 3 miRNAs (hsa-miR-143-3p, hsa-miR-4301, and hsa-miR-548aa) were found (Figure 4(a)), of which only miR-143-3p had a significantly higher expression in ATC patients than in normal controls (Figure 4(b)). Therefore, we chose miR-143-3p as the target gene for follow-up research.

As reflected by RT-qPCR, miR-143-3p expression in the peripheral blood of ATC patients and ATC mice increased significantly (Figures 4(c) and 4(d)). To further explore the regulatory relationship between miR-143-3p and VWF, we predicted through the StarBase database that there was a binding site between miR-143-3p and VWF (Figure 4(e)). The results of luciferase experiment confirmed that compared with the NC group, the luciferase activity in the cells cotransfected with miR-143-3p and VWF-WT was significantly reduced, but no significant effect was observed on the activity of the VWF-MUT reporter gene (Figure 4(f)).

In addition, miR-143-3p mimic or miR-143-3p inhibitor was transfected into HUVECs. It was evident that miR-143-3p mimic treatment elevated miR-143-3p expression but reduced VWF expression, while miR-143-3p inhibitor treatment led to opposite trends (Figures 4(g) and 4(h)). The above results indicated that miR-143-3p could target and inhibit VWF.

### 3.5. miR-143-3p Aggravates Coagulation Dysfunction in ATC Mice by Targeting VWF

**Figure 5:**
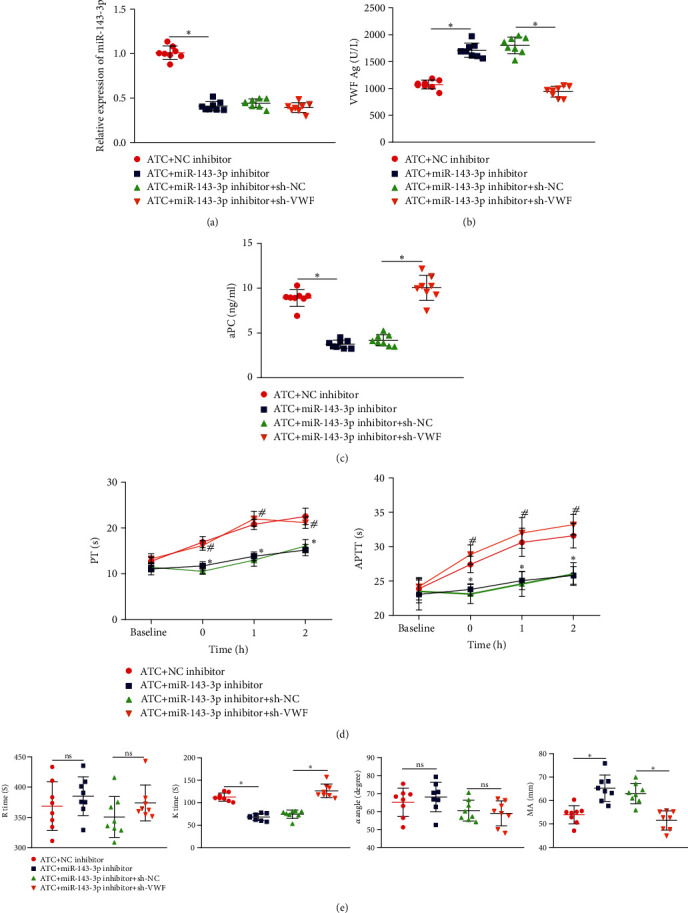
miR-143-3p leads to coagulation dysfunction in ATC mice by inhibiting VWF. (a) RT-qPCR to detect the expression of miR-143-3p in the peripheral blood of each group of mice. (b) ELISA to detect the expression of VWF in the peripheral blood of each group of mice. (c) ELISA to detect aPC in the peripheral blood of each group of mice. (d) Comparison of prothrombin time and activated partial thromboplastin time in each group of mice (^∗^*p* < 0.05, ATC+miR-143-3p inhibitor+sh-NC; ^#^*p* < 0.05, ATC+NC-inhibitor). (e) Comparison of TEG test results of each group of mice. *n* = 8 mice for each group. ^∗^*p* < 0.05.

We then moved to explore the role of miR-143-3p regulating VWF in coagulation function. A constructed ATC mouse model was injected with NC inhibitor, miR-143-3p inhibitor, miR-143-3p inhibitor, sh-NC, or sh-VWF. We found from RT-qPCR that ATC mice injected with miR-143-3p inhibitor showed decreased miR-143-3p expression, while further injection of sh-VWF caused no significant change in miR-143-3p expression (Figure 5(a)). Additionally, ATC mice injected with miR-143-3p inhibitor had increased VWF expression in peripheral blood, while further injection of sh-VWF reduced VWF expression in peripheral blood (Figure 5(b)). After 1 h of trauma, the aPC concentration in the peripheral blood of ATC mice injected with miR-143-3p inhibitor was significantly lower, while further injection of sh-VWF led to opposite trends (Figure 5(c)). We also found decreased PT and APTT in ATC mice injected with miR-143-3p inhibitor, while ATC mice injected with miR-143-3p inhibitor and sh-VWF had elevated PT and APTT (Figure 5(d)). Observation from TEG indicated that ATC mice injected with miR-143-3p inhibitor had decreased *K* time but elevated MA, and no significant difference was observed in *R* time and *α* angle, while ATC mice injected with miR-143-3p inhibitor and sh-VWF had elevated *K* time but decreased MA, and no significant difference was observed in *R* time and *α* angle (Figure 5(e)). These results showed that miR-143-3p could aggravate coagulation dysfunction in ATC mice by inhibiting VWF.

### 3.6. circ_0001274 Competitively Binds miR-143-3p to Upregulate VWF Expression

**Figure 6:**
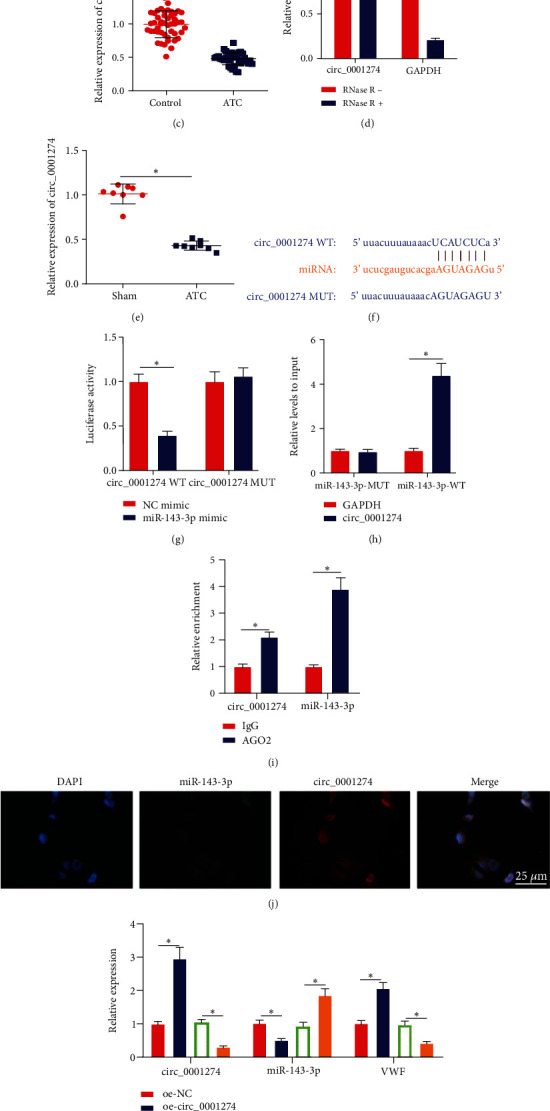
circ_0001274 could competitively bind miR-143-3p to upregulate the expression of VWF. (a) Venn diagram of the circular RNA upstream of miR-143-3p predicted by StarBase database and ATC-related differentially expressed circular RNA. (b) Heat map of ATC-related differentially expressed circular RNA. (c) RT-qPCR detection of level of circ_0001274 in peripheral blood in ATC patients (non-ATC: *n* = 48, ATC: *n* = 39). (d) RNase R was used to determine the stability of circRNA. (e) RT-qPCR to detect the expression of circ_0001274 in the peripheral blood of mice in each group. (f) The target binding site of circ_0001274 and miR-143-3p predicted by StarBase database. (g) Dual luciferase assay to detect the binding of circ_0001274 to miR-143-3p. (h) RNA pull-down to detect the direct binding of circ_0001274 to miR-143-3p. (i) RIP experiment to detect the competitively binding of circ_0001274 and miR-143-3p. (j) FISH experiment to detect the localization of circ_0001274 and miR-143-3p in HUVECs (400×). (k) RT-qPCR to detect the expression of circ_0001274, miR-143-3p, and VWF in the cells of each group. (l) Western blot detection of the protein expression of VWF in the cells of each group. *n* = 8 mice for each group. ^∗^*p* < 0.05.

We used the StarBase database to predict the upstream circRNA of miR-143-3p and screened out 1659 circRNAs. In addition, circRNA sequencing was performed on the peripheral blood samples of 3 ATC patients and 3 normal healthy people, and 527 differentially expressed circular circRNAs were screened. The intersection of the online database prediction results and the high-throughput sequencing results showed that only circ_0001274 was obtained (Figure 6(a)), and circ_0001274 was significantly lowly expressed in the peripheral blood of ATC patients (Figure 6(b)). Therefore, we speculated that the circ_0001274/miR-143-3p/VWF regulatory network may be involved in the occurrence of ATC.

For validation, RT-qPCR detection of circ_0001274 expression in the peripheral blood of ATC patients revealed that circ_0001274 expression in the peripheral blood of ATC patients was lower than that of non-ATC patients (Figure 6(c)). The RNase R digestion results showed that the GAPDH content decreased significantly after RNase R digestion, while the content of circ_0001274 was not significantly different from that before RNase R digestion, indicating that circ_0001274 was a circular RNA (Figure 6(d)). In addition, circ_0001274 expression in the peripheral blood of ATC mice was lower than that in the sham-operated mice (Figure 6(e)).

To further explore the regulatory relationship between circ_0001274 and miR-143-3p, we first predicted through the StarBase database that there were binding sites between circ_0001274 and miR-143-3p (Figure 6(f)). Luciferase experiment confirmed that compared with the NC mimic group, the luciferase activity in the cells cotransfected with miR-143-3p mimic and circ_0001274 WT was significantly reduced, but there was no difference in the circ_0001274 MUT group, indicating that circ_0001274 could target miR-143-3p (Figure 6(g)). In addition, RNA pull-down results showed that compared with miR-143-3p-MUT, miR-143-3p-WT was enriched more with circ_0001274 (Figure 6(h)). The results of the RIP experiment showed that AGO2 antibody could simultaneously enrich circ_0001274 and miR-143-3p (Figure 6(i)). FISH experiment found that circ_0001274 and miR-143-3p were colocalized in the cytoplasm (Figure 6(j)). The above results suggested that circ_0001274 could target and regulate miR-143-3p.

After transfection of oe-NC, oe-circ_0001274, sh-NC, or sh-circ_0001274 into HUVECs, we found that oe-circ_0001274 treatment led to elevated expression of circ_0001274 and VWF but reduced miR-143-3p while sh-circ_0001274 transfection caused opposite trends (Figures 6(k) and 6(l)). The above results concluded that circ_0001274 could competitively bind miR-143-3p to upregulate the expression of VWF, and the circ_0001274/miR-143-3p/VWF regulatory network may be a key pathway involved in the occurrence of ATC.

### 3.7. circ_0001274 Improves the Coagulation Function of ATC Mice through Regulation of miR-143-3p-Mediated VWF

**Figure 7:**
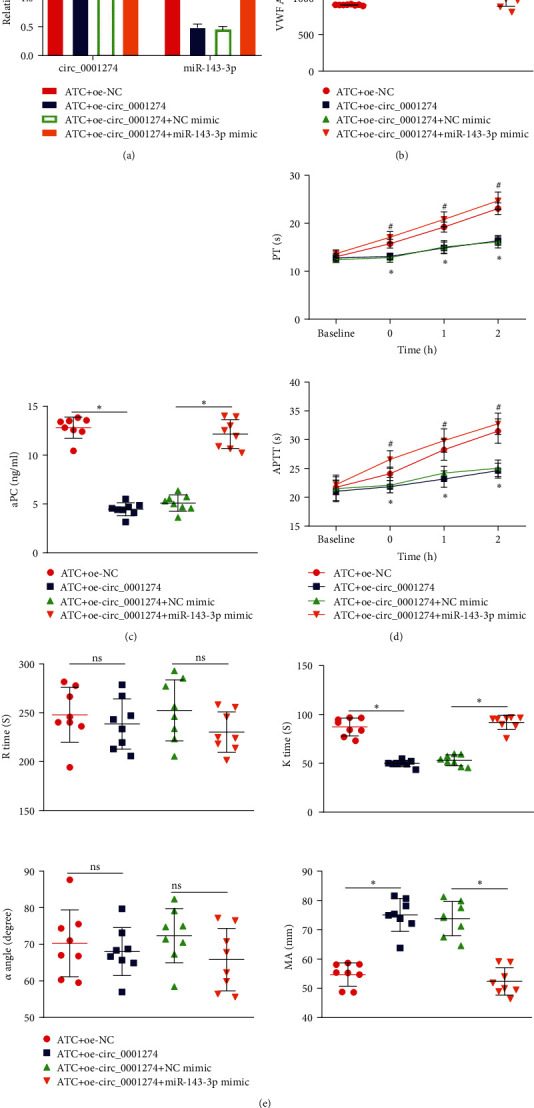
circ_0001274 upregulates VWF by competitively binding miR-143-3p to improve the coagulation function of ATC mice. (a) RT-qPCR to detect the expression of circ_0001274 and miR-143-3p in the peripheral blood of each group of mice. (b) ELISA to detect the expression of VWF in the peripheral blood of each group of mice. (c) ELISA to detect the aPC concentration in peripheral blood of mice in each group. (d) Comparison of prothrombin time and activated partial thromboplastin time in mice of each group. (e) Comparison of TEG test results of mice in each group. *n* = 8 mice for each group. ^∗^*p* < 0.05.

The effect of circ_0001274/miR-143-3p/VWF regulatory network on the occurrence of ATC was then investigated. RT-qPCR determination revealed that ATC mice injected with oe-circ_0001274 had increased circ_0001274 expression but reduced miR-143-3p expression while further injection of miR-143-3p mimic led to upregulated miR-143-3p expression (Figure 7(a)). ELISA showed that ATC mice injected with oe-circ_0001274 had increased VWF expression while further injection of miR-143-3p mimic led to reduced VWF expression (Figure 7(b)). After 1 h of trauma, the aPC concentration in the peripheral blood of ATC mice injected with oe-circ_0001274 was significantly lower, while further injection of miR-143-3p mimic led to opposite trends (Figure 7(c)). We also found decreased PT and APTT in ATC mice injected with oe-circ_0001274, while ATC mice injected with oe-circ_0001274 and miR-143-3p mimic had elevated PT and APTT (Figure 7(d)). Observation from TEG indicated that ATC mice injected with oe-circ_0001274 had decreased *K* time but elevated MA, and no significant difference was observed in *R* time and *α* angle, while ATC mice injected with oe-circ_0001274 and miR-143-3p mimic had elevated *K* time but decreased MA, and no significant difference was observed in *R* time and *α* angle (Figure 7(e)). These results demonstrated that circ_0001274 improves the coagulation function of ATC mice through regulation of miR-143-3p-mediated VWF.

## 4. Discussion

Recent research has focused on the implications of circRNAs in the pathology, pathophysiology, and pathogenesis of diseases and the underlying mechanisms of action [24]. This study surprisingly revealed that circ_0001274 could improve the coagulation activity, whereby protecting against ATC. Furthermore, a circRNA-miRNA-mRNA regulatory network wherein circ_0001274 upregulated VWF by competitively binding miR-143-3p was demonstrated in the development of ATC.

First of all, our study found decreases of VWF in the peripheral blood of ATC patients and mice modeling ATC as well as a beneficial effect of VWF on the coagulation function. VWF is a known acute phase reactant rapidly secreted upon injury which is related to endothelial activation [25–27]. An existing study has reported that an antibody against the VWF A1 domain can protect against inflammation-induced vascular leakage [28]. Endothelial VWF enhances blood-brain barrier flexibility and protects against hypoxia and seizures in mice [29]. Moreover, VWF-bound microvesicles are reported as contributors to promotion of systemic coagulation [30]. Endothelial cells, platelets, VWF, and coagulation factors have shown complex interactions during the haemostatic process which stops bleeding at the vascular injury area and retains the blood vessel integrity by forming clots [31]. It has been suggested that VWF can bind to platelets and collagen, whereby promoting coagulation at sites of injury [32]. Previous studies have widely revealed the crucial hemostatic roles of VWF and its mechanism of action. VWF can recruit platelets to damaged area and act as a protective transporter for coagulation factor VIII (FVIII) via preventing degradation and enhancing stabilization of FVIII [7, 9, 33]. In addition, the premature loss of FVIII caused by the VWF deficiency may induce the dual defect in haemostasis in the patients with von Willebrand disease (VWD). Hence, targeting the VWF deficiency may aid in the protection against VWD through correcting the associated defect in FVIII activity [34]. More recently, an imbalance of VWF/ADAMTS13 is demonstrated to correlate with poor outcome and bleeding [35]. These researches have widely indicated the beneficial role of VWF in the haemostasis, which is consistent with our results as evidenced by reductions in PT and APTT values and *K* time upon restoration of VWF. Endothelial activation of protein C leads to rapid anticoagulation and fibrinolysis responding to severe trauma [36]. It has been shown that an *in vitro* addition of aPC may trigger a phenotype of ATC in healthy blood, suggestive of its vital role in coagulation abnormalities [37]. Moreover, VWF and FVIII contribute to resistance to dilutional coagulopathy and aPC in normal gestational women [38]. It has been shown that aPC inactivates activated FVIII by proteolytic cleavage at Arg336 in the presence of negatively charged phospholipids; that is, APC-catalyzed FVIII inactivation is PL-dependent, and VWF prevents FVIII from interacting with PL, thereby protecting FVIII from various PL-dependent proteases [39–41]. VWF can directly inhibit the binding of aPC to FVIII to protect FVIII from aPC catalysis [40]. Consistent with these findings, this study further provided evidence that *in vivo* overexpression of VWF reduced the aPC level. Hence, it is rational to conclude that VWF may improve coagulation function through inhibition of aPC.

Another important finding was that circ_0001274 positively regulated the expression of VWF by acting as a ceRNA of miR-143-3p. An existing study has mentioned the involvement of miR-143 in hemostatic processes [42]. It has been reported that miR-143 functions as a strong modulator of vascular morphology and that miR-143 can mediate the cerebral vasculature in a rat model of subarachnoid hemorrhage [43]. Also, miR-143-3p has been documented as one of the downregulated EV-miRNAs in response to PM10 exposure, which is related to enhanced coagulation [44]. Besides, the interaction between miR-143 and VWF has been unraveled in an lncRNA-miRNA-mRNA network. It has illustrated that miR-143 overexpression can impair TUG1-induced restoration of VWF [45]. circ_0001274 is a differentially expressed circRNA in the fetal side of placenta from maternal polycystic ovary syndrome [16]. However, the involvement of circ_0001274 in human diseases remains largely unknown, especially in coagulation disorders. circRNAs are a class of RNA transcripts that are circular RNAs which can function as ceRNAs or natural miRNA sponges to involve in the posttranscriptional regulation of gene expression [46]. According to our study, manipulation of miRNA axis may lead to partial reduction of VMP rather than total reduction. It is speculated that there may be other reasons for the downregulation of VMP expression in ATC. Previous study has shown that KCNQ1OT1 and hsa-miR-24-3p have been confirmed to be key upstream regulators of VWF. When KCNQ1OT1 changes, the binding of hsa-miR-24-3p and VWF may be affected, thus affecting the expression of VMP [20]. In addition, although exogenous VWF significantly reduces aPC levels and improves coagulation function in ATC mice, it did not restore aPC to baseline levels (Figure 3(c)), which was also due to the regulation of other miRNA axes [40]. In this study, we identified circ_0001274 as a ceRNA of miR-143-3p to regulate the posttranscriptional level of VWF. Moreover, *in vivo* experimental results demonstrated that circ_0001274 promoted the coagulation function in ATC mice by blocking miR-143-3p-mediation inhibition of VWF.

## 5. Conclusion

**Figure 8:**
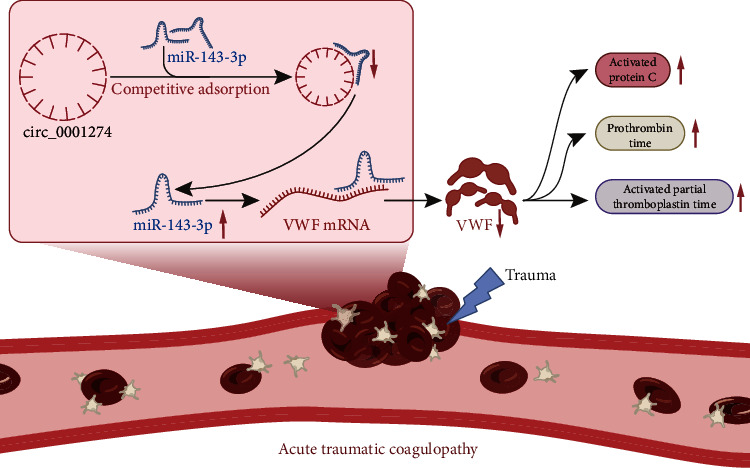
Schematic map of the circ_0001274/miR-143-3p/VWF regulatory network in the occurrence of ATC. Acute trauma leads to the downregulation of circ_0001274, which reduces the competitive adsorption of miR-143-3p, resulting in the increase of free miR-143-3p. The free miR-143-3p can bind more VWF mRNA to reduce the expression of VWF, thus triggering ATC.

Taken together, this study uncovered the involvement of a circ_0001274/miR-143-3p/VWF regulatory network in the occurrence of ATC and suggested the promising protective role of circ_0001274 against coagulation disorders (Figure 8). These findings contribute to the development of new hemostatic strategies which warrant more in-depth investigation.

## Data Availability

The datasets generated/analyzed during the current study are available.

## Supplementary Materials

Supplementary Materials Table S1: comparison of indicators of patients in the two groups. Table S2: the meaning of TEG indicators. Table S3: transfection sequence information. Table S4: RT-qPCR primer sequences.

## References

[1] Kushimoto S., Kudo D., Kawazoe Y. (2017). Acute traumatic coagulopathy and trauma-induced coagulopathy: an overview. *Journal of Intensive Care*.

[2] Meledeo M. A., Herzig M. C., Bynum J. A. (2017). Acute traumatic coagulopathy. *Journal of Trauma and Acute Care Surgery*.

[3] Frith D., Davenport R., Brohi K. (2012). Acute traumatic coagulopathy. *Current Opinion in Anaesthesiology*.

[4] Simmons J. W., Powell M. F. (2016). Acute traumatic coagulopathy: pathophysiology and resuscitation. *British Journal of Anaesthesia*.

[5] Curry N., Stanworth S., Hopewell S., Dorée C., Brohi K., Hyde C. (2011). Trauma-Induced Coagulopathy–A Review of the Systematic Reviews: Is There Sufficient Evidence to Guide Clinical Transfusion Practice?. *Transfusion Medicine Reviews*.

[6] Lenting P. J., Casari C., Christophe O. D., Denis C. V. (2012). Von Willebrand factor: the old, the new and the unknown. *Journal of Thrombosis and Haemostasis*.

[7] Denis C. V., Lenting P. J. (2012). Von Willebrand factor: at the crossroads of bleeding and thrombosis. *International Journal of Hematology*.

[8] Yasar S. J., Abdullah O., Fay W., Balla S. (2018). Von Willebrand factor revisited. *Journal of Interventional Cardiology*.

[9] Randi A. M., Smith K. E., Castaman G. (2018). Von Willebrand factor regulation of blood vessel formation. *Blood*.

[10] Jankowska K. I., Sauna Z. E., Atreya C. D. (2020). Role of micrornas in hemophilia and thrombosis in humans. *International Journal of Molecular Sciences*.

[11] Rangrez A. Y., Massy Z. A., Metzinger-Le Meuth V., Metzinger L. (2011). miR-143 and miR-145. *Circulation: Cardiovascular Genetics*.

[12] Vacante F., Denby L., Sluimer J. C., Baker A. H. (2019). The function of miR-143, miR-145 and the miR-143 host gene in cardiovascular development and disease. *Vascular Pharmacology*.

[13] Hansen T. B., Jensen T. I., Clausen B. H. (2013). Natural RNA circles function as efficient microRNA sponges. *Nature*.

[14] Rybak-Wolf A., Stottmeister C., Glažar P. (2015). Circular RNAs in the mammalian brain are highly abundant, conserved, and dynamically expressed. *Molecular Cell*.

[15] Alhasan A. A., Izuogu O. G., Al-Balool H. H. (2016). Circular RNA enrichment in platelets is a signature of transcriptome degradation. *Blood*.

[16] Zhao C., Zhou Y., Shen X. (2020). Circular RNA expression profiling in the fetal side of placenta from maternal polycystic ovary syndrome and circ_0023942 inhibits the proliferation of human ovarian granulosa cell. *Archives of Gynecology and Obstetrics*.

[17] Linn S. (1995). The injury severity score--Importance and uses. *Annals of Epidemiology*.

[18] Chesebro B. B., Rahn P., Carles M. (2009). Increase in activated protein C mediates acute traumatic coagulopathy in mice. *Shock*.

[19] Frith D., Goslings J. C., Gaarder C. (2010). Definition and drivers of acute traumatic coagulopathy: clinical and experimental investigations. *Journal of Thrombosis and Haemostasis*.

[20] Luo Y., Fu Y., Tan T. (2022). Screening of lncRNA-miRNA-mRNA coexpression regulatory networks involved in acute traumatic coagulation dysfunction based on CTD, GeneCards, and PharmGKB databases. *Oxidative Medicine and Cellular Longevity*.

[21] Zhao H., He Y. (2021). The inhibitory effect of lysophosphatidylcholine on proangiogenesis of human CD34(+) cells derived endothelial progenitor cells. *Frontiers in Molecular Biosciences*.

[22] Li B., Zhou Y., Chen J. (2021). Long noncoding RNA h19 acts as a miR-29b sponge to promote wound healing in diabetic foot ulcer. *The FASEB Journal*.

[23] Zeineddin A., Dong J. F., Wu F., Terse P., Kozar R. A. (2021). Role of von Willebrand factor after injury: it may do more than we think. *Shock*.

[24] Han B., Chao J., Yao H. (2018). Circular RNA and its mechanisms in disease: from the bench to the clinic. *Pharmacology & Therapeutics*.

[25] Yokota H., Naoe Y., Nakabayashi M. (2002). Cerebral endothelial injury in severe head injury: the significance of measurements of serum thrombomodulin and the von Willebrand factor. *Journal of Neurotrauma*.

[26] Yokota H. (2007). Cerebral endothelial damage after severe head injury. *Journal of Nippon Medical School*.

[27] Akyol O., Akyol S., Chen C. H. (2016). Update on ADAMTS13 and VWF in cardiovascular and hematological disorders. *Clinica Chimica Acta*.

[28] Aymé G., Adam F., Legendre P. (2017). A novel single-domain antibody against von Willebrand factor a1 domain resolves leukocyte recruitment and vascular leakage during inflammation–brief report. *Arteriosclerosis, Thrombosis, and Vascular Biology*.

[29] Suidan G. L., Brill A., De Meyer S. F. (2013). Endothelial von Willebrand factor promotes blood-brain barrier flexibility and provides protection from hypoxia and seizures in mice. *Arteriosclerosis, Thrombosis, and Vascular Biology*.

[30] Wu Y., Liu W., Zhou Y. (2018). Von Willebrand factor enhances microvesicle-induced vascular leakage and coagulopathy in mice with traumatic brain injury. *Blood*.

[31] Sang Y., Roest M., de Laat B., de Groot P. G., Huskens D. (2021). Interplay between platelets and coagulation. *Blood Reviews*.

[32] Flood V. H., Slobodianuk T. L., Keesler D. (2020). Von Willebrand factor binding to myosin assists in coagulation. *Blood Advances*.

[33] Ruggeri Z. M. (2007). Von Willebrand factor: looking back and looking forward. *Thrombosis and Haemostasis*.

[34] Miesbach W., Berntorp E. (2015). Interaction between VWF and FVIII in treating VWD. *European Journal of Haematology*.

[35] Driever E. G., Stravitz R. T., Zhang J. (2021). VWF/ADAMTS13 imbalance, but not global coagulation or fibrinolysis, is associated with outcome and bleeding in acute liver failure. *Hepatology*.

[36] Davenport R. (2013). Pathogenesis of acute traumatic coagulopathy. *Transfusion*.

[37] Howard B. M., Kornblith L. Z., Cheung C. K. (2016). Inducing acute traumatic coagulopathy in vitro: the effects of activated protein c on healthy human whole blood. *PLoS One*.

[38] Tanaka K. A., Bharadwaj S., Hasan S. (2019). Elevated fibrinogen, von Willebrand factor, and factor viii confer resistance to dilutional coagulopathy and activated protein C in normal pregnant women. *British Journal of Anaesthesia*.

[39] Nogami K., Shima M., Nishiya K. (2002). A novel mechanism of factor VIII protection by von Willebrand factor from activated protein C-catalyzed inactivation. *Blood*.

[40] Ammollo C. T., Semeraro F., Vitulli A. (2020). FVIII/VWF complex displays a greater pro-haemostatic activity than FVIII preparations devoid of VWF: study in plasma and cell-based models. *Haemophilia*.

[41] Koedam J. A., Meijers J. C., Sixma J. J., Bouma B. N. (1988). Inactivation of human factor VIII by activated protein C. cofactor activity of protein S and protective effect of von Willebrand factor. *The Journal of Clinical Investigation*.

[42] Stachowiak G., Zajac A., Nowak M., Stetkiewicz T., Wilczynski J. R. (2015). Hemostatic disorders of the menopausal period: the role of microRNA. *Menopausal Review*.

[43] Bai Y., Zhang Y., Han B. (2018). Circular RNA DLGAP4 ameliorates ischemic stroke outcomes by targeting miR-143 to regulate endothelial-mesenchymal transition associated with blood-brain barrier integrity. *The Journal of Neuroscience*.

[44] Pergoli L., Cantone L., Favero C. (2017). Extracellular vesicle-packaged miRNA release after short-term exposure to particulate matter is associated with increased coagulation. *Particle and Fibre Toxicology*.

[45] Xue Y. N., Yan Y., Chen Z. Z. (2019). lncRNA TUG1 regulates FGF1 to enhance endothelial differentiation of adipose- derived stem cells by sponging miR-143. *Journal of Cellular Biochemistry*.

[46] Tay Y., Rinn J., Pandolfi P. P. (2014). The multilayered complexity of ceRNA crosstalk and competition. *Nature*.

