# Supplementary material for: circ_0001274 Competitively Binds miR-143-3p to Upregulate VWF Expression to Improve Acute Traumatic Coagulopathy
Source: Oxid Med Cell Longev. 2023 Jan 31;2023:9650323. doi: 10.1155/2023/9650323 (PMC9904904; doi:10.1155/2023/9650323)
Supplement: Supplementary Materials — Table S1: comparison of indicators of patients in the two groups. Table S2: the meaning of TEG indicators. Table S3: transfection sequence information. Table S4: RT-qPCR primer sequences. [file 9650323.f1.docx]

**Table S1** Comparison of indicators of patients in the two groups

| Clinical indicators | Non-ATC (n = 48) | ATC (n = 39) | *p* |
| --- | --- | --- | --- |
| Age | 42.8 ± 6.6 | 40.6 ± 5.8 | <0.001* |
| Gender |  |  | 0.879 |
| Man, n (%) | 35 (72.9) | 29 (74.4) |  |
| Women, n (%) | 13 (27.1) | 10 (25.6) |  |
| Injury mechanism |  |  | 0.985 |
| Accident, n (%) | 23 (47.9) | 20 (51.3) |  |
| Falling from a tall building, n (%) | 10 (20.8) | 9 (23.1) |  |
| Fall, n (%) | 7 (44.3) | 5 (12.8) |  |
| Sharp injury, n (%) | 5 (10.4) | 3 (7.7) |  |
| Other, n (%) | 3 (6.2) | 2 (5.1) |  |
| Arrival time, h | 5.9 ± 1.6 | 5.4 ± 1.2 | 0.101 |
| Injured part |  |  |  |
| Head, n (%) | 35 (64.9) | 31 (71.2) |  |
| Face, n (%) | 8 (12.3) | 5 (14.3) |  |
| Cheat, n (%) | 33 (57.9) | 30 (73.5) |  |
| Abdomen, n (%) | 14 (21.1) | 11 (22.4) |  |
| Limbs and pelvis, n (%) | 28 (49.1) | 22 (44.9) |  |
| Body surface, n (%) | 25 (38.6) | 20 (38.8) |  |
| ISS scale | 20.5 ± 8.4 | 23.7 ± 8.7 | 0.091 |
| Temperature, ℃ | 36.4 ± 0.5 | 36.2 ± 0.7 | 0.077 |
| Pulse, time/min | 83.8 ± 10.9 | 95. ± 17.7 | 0.001* |
| Respiratory rate, time/min | 15.8 ± 2.8 | 14.7 ± 2.9 | 0.092 |
| Systolic blood pressure, mmHg | 127.6 ± 20.4 | 119.6 ± 23.2 | 0.092 |
| Diastolic blood pressure, mmHg | 76.4 ± 14.7 | 70.7 ± 13.5 | 0.066 |
| Hemoglobin concentration, g/L | 135.9 ± 18.6 | 129.3 ± 16.5 | 0.088 |
| Platelet count, 10^9^/L | 187.6 ± 65.5 | 164.7 ± 44.0 | 0.066 |
| Plasma prothrombin time (s) | 13.4 ± 2.1 | 17.2 ± 3.6 | <0.001* |
| Activated partial thromboplastin time (s) | 30.5 ± 5.4 | 40.2 ± 5.6 | <0.001* |
| International standard ratio | 0.98 ± 0.05 | 1.40 ± 0.10 |  |
| Fibrinogen concentration, g/L | 2.67 ± 0.49 | 2.06 ± 0.33 | <0.001* |
| VWF antibody (U/L) | 1503.8 ± 288.4 | 1108.9 ± 208.1 | <0.001* |

**Table S2** The meaning of TEG indicators

| Indicators | unit | clinical significance |
| --- | --- | --- |
| reaction time, R time | s | From the beginning of the test until the thrombus amplitude reaches 2 mm, it represents the initiation process of procoagulant factor zymogen |
| coagulation time, K time | s | The coagulation factor is activated until the thrombus amplitude reaches 20 mm, reflecting the rate at which thrombin promotes fibrin production |
| α angle | ° | The tangent angle of the R time--K time curve, reflecting the speed of thrombus formation |
| maximum amplitude, MA | mm | The maximum amplitude of the TEG curve indicates that platelets and fibrin have the strongest effect on thrombus formation through the GPIIb-IIIa receptor |

**Table S3** Transfection sequence information

| Name | Sequence |
| --- | --- |
| oe-NC | 5’-AGTCGCGATGCGATGCCCCGTACGAC-3’ |
| oe-circ_0001274 | 5’-GATGGTACAAAACAGAAGAGGGAACGGAAAAAGACAGTCTCATTCAGCAGCATGCCAACAGAGAAGAAGATCAGCAGTGCAAGTGATTGTATTAATTCAATGGTTGAGGGTTCAGAACTCAAAAAGGTTCGCTCCAACTCTAGAATTTATCATAGGTACTTTTTACTGGATGCTGACATGCAGAGCCTAAGGTGGGAGCCATCTAAGAAGGATTCTGAGAAAGCCAAGATTGACATTAAATCCATCAAGGAAGTGAGAACAGGAAAAAACACAGACATATTCCGCAGCAATGGCATTTCTGACCAGATATCTGAAGATTGTGCGTTTTCCGTCATATATGGAGAGAATTATGAGTCACTGGATTTGGTTGCCAACTCCGCAGATGTTGCAAACATCTGGGTTACAGGACTGCGGTACCTAATTTCTTATGGAAAACATACACTTGATATGTTAGAAAGTAGCCAAGATAACATGAGGACTTCTTGGGTTTCACAAATGTTTAGTGAAATTGATGTAGATAACCTTGGACATATAACTCTGTGTAATGCTGTGCAATGTATCAGAAACCTCAATCCTGGTTTAAAAACGAGCAAAATTGAGCTTAAGTTCAAAGAATTGCATAAATCAAAGGACAAAGCTGGTACCGAGGTCACAAAGGAAGAATTTATTGAGGTTTTTCATGAGCTTTGTACTAGACCTGAAATTTATTTCCTTTTAGTTCAGTTTTCAAGCAATAAAGAATTCCTTGATACCAAGGACCTTATGATGTTTCTTGAGGCAGAACAGGGTGTGGCACATATAAATGAGGAAATAAGCCTTGAAATTATTCACAAATATGAACCATCCAAAGAGGGTCAGGAAAAGGGCTGGCTCTCCATAGACGGGTTCACTAATTACCTTATGTCACCTGACTGTTATATATTCGATCCAGAACATAAGAAGGTCTGTCAGGATATGAAGCAACCTCTGTCTCATTACTTTATAAACTCATCTCATAATACATACTTAATAGAGGATCAGTTCCGAGGTCCCTCCGACATCACAGGATATATTCGAGCTCTTAAAATGGGTTGCCGGAGTGTTGAATTAGATGTATGGGATGGGCCGGACAATGAACCTGTAATTTACACAGGCCACACCATGACCTCTCAGATAGTTTTCCGCAGTGTCATTGATATTATTAACAAGTATGCATTCTTTGCTTCAGAGTATCCTCTTATCTTGTGTTTAGAAAACCACTGTTCCATTAAACAACAGAAGGTAATGGTTCAGCACATGAAGAAACTTTTAGGAGACAAGCTCTATACAACATCACCCAATGTTGAGGAATCTTATCTACCATCCCCAGATGTCCTGAAAGGGAAAATACTAATTAAAGCAAAGAAGCTGTCCTCAAATTGCTCTGGGGTAGAAGGAGATGTTACTGACGAAGATGAAGGAGCAGAAATGTCTCAGAGGATGGGAAAAGAGAACATGGAGCAACCCAATAATGTGCCTGTGAAGCGATTTCAGCTTTGTAAAGAACTGTCTGAACTGGTCAGCATCTGCAAATCAGTTCAGTTCAAAGAATTTCAGGTGTCGTTTCAGGTTCAGAAGTACTGGGAAGTCTGTTCCTTTAATGAAGTGCTTGCCAGCAAGTACGCCAATGAAAATCCAGGGGACTTTGTAAATTACAACAAACGTTTTCTTGCTAGGGTTTTTCCCAGTCCAATGAGAATTGATTCCAGTAACATGAATCCTCAAGATTTTTGGAAATGTGGTTGCCAAATTGTAGCCATGAACTTTCAGACACCAGGACTGATGATGGACCTGAATATTGGCTGGTTTAGGCAGAACGGAAACTGTGGCTATGTCCTCCGGCCAGCCATCATGAGGGAGGAGGTCTCCTTCTTCAGCGCCAATACAAAAGACTCTGTCCCAGGGGTCTCACCTCAACTTCTTCACATTAAAATCATCAGTGGGCAGAACTTTCCCAAGCCCAAAGGATCAGGTGCCAAAGGTGATGTGGTAGATCCTTATGTCTATGTTGAAATCCATGGAATCCCTGCTGATTGTGCAGAACAAAGGACAAAAACAGTGCACCAGAATGGAGACGCTCCCATTTTTGATGAAAGCTTTGAATTTCAAATCAACCTGCCTGAACTGGCCATGGTGCGCTTTGTAGTGCTGGATGATGACTACATTGGGGATGAATTCATCGGCCAGTACACAATTCCCTTTGAATGTTTACAGACGGGCTACCGCCATGTCCCCCTGCAGTCCTTAACTGGAGAGGTCCTTGCACATGCTTCTTTATTTGTCCACGTGGCTATTACTAACCGAAGAGGAGGAGGAAAGCCTCATAAAAGGGGCCTTTCTGTGAGAAAAGGGAAGAAATCCAGGGAATATGCATCTTTGAGAACACTGTGGATTAAAACCGTGGATGAGGTATTCAAGAATGCCCAGCCCCCTATACGGGATGCCACAGATCTGAGAGAAAACATGCAGAATGCGGTGGTGTCATTCAAGGAGCTGTGTGGCCTCTCCTCTGTGGCCAATCTCATGCAGTGCATGTTGGCGGTGTCTCCCCGCTTTCTGGGGCCCGATAACACACCCCTAGTGGTCCTAAATCTCAGCGAGCAGTACCCCACAATGGAGCTGCAGGGAATTGTGCCGGAGGTTCTGAAGAAGATCGTAACAACTTATGACATG-3’ |
| sh-NC | 5’-CATGCAGTGCAGCGACGAGCA-3’ |
| sh-circ_0001274 | 5’-GCGAGCAGTACCCCACAATG-3’ |
| mimic-NC | Forward: 5’-UUCUCCGAACGUGUC-3' |
| miR-143-3p mimic | Forward: 5’-UGAGAUGAAGCACUGUAGCUC-3' |
| inhibitor-NC | Forward: 5'-CAGUACUUUUGUGUAGUAAA-3' |
| miR-143-3p inhibitor | Forward: 5'-GAGCUACAGUGCUUCAUCUCA-3' |

**Table S4** RT-qPCR primer sequences

| Gene | Sequence |
| --- | --- |
| circ_0001274 (*mouse*) | Forward: 5ʹ-TCTGGCTGAACGGATTCGAG-3’  Reverse: 5ʹ-CACCATCTCGTTCTGCTGGT-3’ |
| circ_0001274 (*human*) | Forward: 5ʹ-GCGAGCAGTACCCCACAATG-3’  Reverse: 5ʹ-GGCATGCTGCTGAATGAGACT-3’ |
| U6 (*human*) | Forward: 5′-CTCGCTTCGGCAGCACATATACTA-3′  Reverse: Kit universal reverse primer |
| U6 (*mouse*) | Forward: 5 ′-CTCGCTTCGGCAGCACA-3′  Reverse: Kit universal reverse primer |
| miR-143-3p (*mouse*) | Forward: 5′-TGAGATGAAGCACTGTAGCTC-3′  Reverse: Kit universal reverse primer |
| miR-143-3p (*human*) | Forward: 5′-TGAGATGAAGCACTGTAGCTC-3′  Reverse: 5′-TGGTGTCGTGGAGTCG-3′ |
| VWF (*human*) | Forward: 5′-CCCCTGAAGCCCCTCCTCCTA-3′  Reverse: 5′-ACGAACGCCACATCCAGAACC-3′ |
| GAPDH (*mouse*) | Forward: 5′-CTCAAGATTGTCAGCAATG-3′  Reverse: 5′-GTCATGAGCCCTTCCACA-3′ |
| GAPDH (*human*) | Forward: 5′-AGAAGGCTGGGGCTCATTTG-3′  Reverse: 5′-GCAGGAGGCATTGCTGATGAT-3′ |
